# Positive effects of duckweed polycultures on starch and protein accumulation

**DOI:** 10.1042/BSR20160158

**Published:** 2016-09-16

**Authors:** Yang Li, Fantao Zhang, Maurycy Daroch, Jie Tang

**Affiliations:** * School of Environment and Energy, Peking University Shenzhen Graduate School, Shenzhen, 518055, China; † College of Life Sciences, Jiangxi Normal University, Nanchang, 330022, China; ‡ School of Pharmacy and Bioengineering, Chengdu University, Chengdu, 610106, China

**Keywords:** biomass, duckweed, polyculture, protein, starch

## Abstract

The effect of duckweed species composition (*Lemna aequinoctialis* 5505, *Landoltia punctata* 5506 and *Spirodela polyrhiza* 5507) in polyculture and monoculture on biomass and starch/protein content were investigated at different levels of temperature, light intensity, nitrogen and phosphorus concentrations. The three growth parameters significantly affect duckweed biomass accumulation. Different combinations of duckweed species greatly varied in starch/protein content. Although all the polycultures showed a median relative growth rate and the majority of the polycultures showed a median and starch/protein content as compared with their respective monocultures, some of the polycultures were found to promote the accumulation of starch/protein at different growth conditions. These findings indicated that proper combination of duckweed species could facilitate desirable biomass accumulation and improve biomass quality. The present study provides useful references for future large-scale duckweed cultivation.

## INTRODUCTION

The Lemnaceae (commonly called duckweed) is an aquatic plant that has shown considerable potential in wastewater treatment [1,2]. Duckweed can assimilate nutrients from wastewater and convert them into valuable biomass, primarily composed of starch and protein [3,4]. Under suitable growth conditions, duckweed doubles its biomass in 1–3 days and produces a continued biomass supply for 9–12 months annually [5]. By extrapolating from field-study results, biomass yields of 39.1–105.9 t·ha^−1^·year^−1^ (dry biomass) could be achieved for duckweed using wastewater as a nutrient source, exhibiting substantially higher yields than most other potential energy crops [2,6]. Therefore, duckweed is a promising feedstock for various applications including biofuels and animal feed, given its multiple desirable traits in biomass accumulation and wastewater purification.

The duckweed family consists of five genera: *Spirodela*, *Landoltia*, *Lemna*, *Wolffia* and *Wolffiella*, comprising about 37 different species [7]. Depending on duckweed species and cultivation conditions, the starch contents of duckweed can vary from 3% to 75% of dry weight, while the protein contents from 15% to 45% [8,9]. Moreover, various geographical isolates within species also showed dramatic differences in capabilities of producing biomass [1,10]. Thus, screening desirable duckweed isolates is crucial for further large-scale applications, especially for establishment of local duckweed cropping system.

In previous studies, biomass production by duckweed was mostly conducted by using only a single species [11–13]. However, it is difficult to maintain a single species thoroughly in artificial systems due to common contamination with other species [14,15]. Besides, it is ubiquitous in natural communities that two or more duckweed species clustered together [16,17], indicating polyculture may be a prevailing type of community for duckweed and facilitate their survival. Nevertheless, it remains largely unknown whether a polyculture of different duckweed species influences biomass production. Although Zhao et al. [18] assessed the biomass and starch content of *Lemna minor* and *Landoltia punctata* in monoculture and their polyculture under different condition settings, protein contents, major component of duckweed biomass, were not discussed. Additionally, the different combination of duckweed species might potentially affect biomass production. Therefore, systematic studies are essential for understanding the influence of duckweed species diversity on biomass productivity and should provide useful guidance for future industrial applications of duckweed as a feedstock.

In the present study, biomass, starch and protein content of three local duckweed isolates (*Lemna aequinoctialis, L. punctata* and *Spirodela polyrhiza*) either in polyculture or as monocultures were investigated under different light intensity, temperature and nutrient concentration. The aim of the present study was to evaluate whether mixed cultivation of duckweed species have positive effect on relative growth rate, starch content and protein content, as compared with a monoculture of duckweed.

## MATERIALS AND METHODS

### Plant material and culture condition

Three duckweed isolates, *L. aequinoctialis* LC33, *L. punctata* LC06 and *S. polyrhiza* LC15, were used as plant materials in the present study. The duckweed were all isolated from Lake Chao, Anhui Province, Eastern China, and identified in our previous study [17]. These isolates were also registered at Rutgers Duckweed Stock Cooperative (RDSC) under the accession numbers of *L. aequinoctialis* 5505, *L. punctata* 5506 and *S. polyrhiza* 5507.

The previously established plants were placed in plastic pot (18 cm ×14 cm ×15 cm) containing one-tenth strength of Hoagland solution (macronutrients: 5.00 mmol·l^−1^ KH_2_PO_4_, 15.00 mmol·l^−1^ KNO_3_, 5.00 mmol·l^−1^ Ca(NO_3_)_2_·4H_2_O and 2.03 mmol·l^−1^ MgSO_4_·7H_2_O; micronutrients: 0.05 mmol·l^−1^ H_3_BO_3_, 0.02 mmol·l^−1^ MnCl_2_·4H_2_O, 0.01 mmol·l^−1^ ZnSO_4_·7H_2_O, 0.01 mmol·l^−1^ CuSO_4_·5H_2_O and 0.01 mmol·l^−1^ Na_2_MoO_4_·2H_2_O; tartaric acid, 0.02 mmol·l^−1^). The pH was adjusted to 5.8 throughout the experiment [19].

### Experimental design

The plants were grown in a controlled climate chamber under a photoperiod of 16-h light (105 μmol·m^−2^·s^−1^; 25°C) and 8-h dark (20°C). The mixed cultures were generated by integrating either two or three of the duckweed species by ratios of 1:1 or 1:1:1. A total of 0.3 initial grams of fresh materials were inoculated to cover the 70% of the water surface with a single layer of fronds [20,21].

The relative growth rate, starch content and protein content of duckweed isolates in polyculture or monoculture were investigated under three different parameters using one-tenth strength of Hoagland solution. Three levels of each parameter were tested: temperature (20, 25 and 30°C); light intensity (30, 75 and 105 μmol·m^−2^·s^−1^); and concentration of N and P (35 mg·N·l^−1^, 15 mg·P·l^−1^; 3.5 mg·N·l^−1^, 1.5 mg·P·l^−1^; and 0 mg·N·l^−1^, 0 mg· P·l^−1^). Each parameter was tested separately with the other parameters constant. The relative growth rate and starch/protein content was determined at the end of 12 days. The distilled water was added to replenish evaporated water every day during the experiments. And the growth solution was renewed every 2 days to keep nutrient levels. All experiments were conducted in triplicate.

### Biomass analysis

The fresh weight of duckweed was measured as described by Bergmann et al. [10]. The fresh fronds were lyophilized for 24 h using a FreeZone system (2.5 Liter Benchtop, Labconco) to measure the dry weight (DW). The relative growth rate of duckweed was calculated as (ln *x*_12_ − ln *x*_0_)/*t* [5], where *x*_12_ is fresh weight of plants grown for 12 days, *x*_0_ is initial fresh weight and *t* is cultivation days.

Approximately 10–15 mg of dry duckweed powder was used to measure the starch content using the method described by Zhao et al. [22]. The starch content was determined using the total sugar content (starch content=glucose content × 0.909) as described by Zhang et al. [23].

Crude Protein was measured using the method described by Markus et al. [24], and the protein content was estimated by N × 6.25, where N is the crude protein [25].

### Statistical analysis

Data were analysed by SPSS Version 19.0 software (SPSS). The *Duncan* test was applied to statistically investigate the differences between polyculture and their monoculture in terms of relative growth rate, starch content and crude protein content. All data presented were means of three replicates, and a significance level of 0.05 was applied.

## RESULTS AND DISCUSSION

### Effect of temperature on duckweed growth

Three duckweed isolates in monoculture or polyculture at different levels of temperature were measured at the end of 12 days to determine the effects of different duckweed species combinations and temperature on plant growth. As shown in Table 1, temperature has an evident impact on duckweed growth in terms of relative growth rates and starch/protein content. In all cases of monoculture or polyculture, the highest relative growth rates and protein content was achieved at 25°C, while the highest starch content was achieved at 20°C (Table 1). This is consistent with previous reports in other duckweed species [18].

**Table 1:** Relative growth rate, starch content and crude protein content of the duckweed in the mixture and monoculture under different temperature The relative growth rate and starch/protein content were measured at 12 days after inoculation. Different lower-case letters in the same column denote significant differences according to *Duncan* test (*P*<0.05).

	Relative growth rate (day^−1^)			Starch content (% DW)			Crude protein content (% DW)		
Culture	20°C	25°C	30°C	20°C	25°C	30°C	20°C	25°C	30°C
*L. aequinoctialis*	0.18±0.0071a	0.19±0.0127a	0.17±0.0039a	13.74±0.6400a	12.49±0.0086a	11.13±0.7211a	27.97±0.0979^b^	32.63±0.2883^b^	25.82±0.3690a
*L. punctata*	0.17±0.0035a	0.19±0.0236a	0.17±0.0032a	15.34b±0.2044^b^	13.40±0.4143^bc^	11.67±0.0600^ab^	26.99±0.2197a	31.91±0.5749a	27.69±0.0998^b^
*S. polyrhiza*	0.16±0.0019a	0.18±0.0029a	0.16±0.0043a	17.18±0.5102^c^	13.97±0.3055^cd^	12.79±0.5442^c^	29.61±0.4492^d^	36.20±0.1729e	30.54±0.1316^de^
*L. aequinoctialis*+*L. punctata*	0.17±0.0097a	0.19±0.0139a	0.17±0.0045a	14.99±0.5644^b^	13.05±0.2795^ab^	11.30±0.3053a	27.34±0.1480a	32.07±0.3029^ab^	26.24±0.6183a
*L. aequinoctialis*+*S. polyrhiza*	0.17±0.0071a	0.19±0.0111a	0.18±0.0063a	15.83±1.0633^b^	13.65±0.6360^bc^	13.04±0.4792^c^	31.29±0.2508e	34.52±0.4379^d^	31.02±0.2356e
*L. punctata*+*S. polyrhiza*	0.17±0.0026a	0.19±0.0213a	0.17±0.0098a	16.15±0.8695^bc^	14.38±0.2863^d^	12.39±0.6114^bc^	28.49±0.4089^c^	31.41±0.2325^c^	28.99±0.1270^c^
*L. aequinoctialis*+*L. punctata*+*S. polyrhiza*	0.17±0.0032a	0.19±0.0062a	0.18±0.0118a	15.95±0.6753^bc^	14.01±0.2801^cd^	12.64±0.0519^bc^	30.12±0.1141e	33.97±0.3384^cd^	30.39±0.2213^d^

The relative growth rates of *L. aequinoctialis*, *L. punctata* and *S. polyrhiza* at the optimal temperature (25°C) was 0.19, 0.19 and 0.18 day^−1^ respectively (Table 1). The relative growth rates of all polycultures were between those of the corresponding duckweed isolates in monoculture, suggesting that polycultures had no advantage of relative growth rate over monocultures at different levels of temperature. This is in accord with the previous study regarding the polyculture of *L. minor* OT and *L. punctata* OT [18].

The starch content in all the combinations decreased as the temperature increased from 20 to 30°C (Table 2). This is in accord with previous findings that low temperature resulted in more starch accumulation [18]. The highest starch content was achieved by *S. polyrhiza* (17.18%) at 20°C. Three polycultures exhibited higher starch contents than those of their monocultures, namely *L. punctata*+*S. polyrhiza* (14.38%, at 25°C), *L. aequinoctialis+L. punctata*+*S. polyrhiza* (14.01%, at 25°C) and *L. aequinoctialis*+*S. polyrhiza* (13.04%, at 30°C). These values, however, are not statistically significant to one another. The rest of polycultures at different levels of temperature showed median starch contents compared with their monocultures. Taken together, mixed cultures did not have a significant advantage over the monoculture in terms of starch content.

**Table 2:** Relative growth rate, starch content and crude protein content of the duckweed in the mixture and monoculture under different light intensity The relative growth rate and starch/protein content were measured at 12 days after inoculation. Different lower-case letters in the same column denote significant differences according to *Duncan* test (*P*<0.05).

	Relative growth rate (day^−1^)			Starch content (% DW)			Crude protein content (% DW)		
	30	75	105	30	75	105	30	75	105
Culture	μmol·m^−2^·s^−1^	μmol·m^−2^·s^−1^	μmol·m^−2^·s^−1^	μmol·m^−2^·s^−1^	μmol·m^−2^·s^−1^	μmol·m^−2^·s^−1^	μmol·m^−2^·s^−1^	μmol·m^−2^·s^−1^	μmol·m^−2^·s^−1^
*L. aequinoctialis*	0.11±0.0320a	0.18±0.0192a	0.19±0.0137a	6.50±0.2533a	11.60±0.2156a	13.15±0.1872a	16.17±0.1414^ab^	29.53±0.2635a	33.73±0.3812^c^
*L. punctata*	0.11±0.0073a	0.17±0.0172a	0.18±0.0092a	7.04±0.3780^ab^	12.94±0.2100^b^	15.48±0.2219^cd^	15.76±0.1220a	30.14±0.2158^b^	32.27±0.2988a
*S. polyrhiza*	0.10±0.0337a	0.16±0.0185a	0.15±0.0101a	7.35±0.2509^bc^	13.84±0.2397^d^	16.28±0.3378e	17.21±0.0691^cd^	32.60±0.1946e	36.82±0.6453e
*L. aequinoctialis*+*L. punctata*	0.12±0.0013a	0.18±0.0059a	0.18±0.0121a	6.78±0.2835^ab^	13.06±0.2218^bc^	15.69±0.5655^de^	16.09±0.0825^ab^	30.09±0.1523^b^	32.89±0.4836^b^
*L. aequinoctialis*+*S. polyrhiza*	0.11±0.0311a	0.18±0.0192a	0.19±0.0093a	6.84±0.3421^ab^	11.81±0.3364a	14.81±0.5229^bc^	16.55±0.3152^bc^	33.00±0.2940^f^	34.52±0.7337^d^
*L. punctata*+*S. polyrhiza*	0.12±0.0165a	0.17±0.0032a	0.18±0.0129a	7.19±0.3022^bc^	13.73±0.3338^cd^	15.85±0.3816^de^	16.16±0.1506^ab^	31.88±0.1286^d^	36.97±0.5011e
*L. aequinoctialis*+*L. punctata*+*S. polyrhiza*	0.11±0.0151a	0.17±0.0154a	0.18±0.0155a	7.71±0.3816^c^	13.07±0.4028^bc^	14.65±0.4061^b^	17.60±0.3799^d^	31.40±0.1343^c^	33.82±0.3626^c^

Similar to the relative growth rates, the highest protein content of *L. aequinoctialis*, *L. punctata* and *S. polyrhiza* was achieved at 25°C, yielding a protein content of 32.63%, 31.91% and 36.20% respectively (Table 2). Interestingly, two polycultures at low temperature had a significant effect on protein accumulation, as compared with their monocultures. The protein content of the polyculture of *L. aequinoctialis* and *S. polyrhiza* at 20°C was 31.29%, significantly higher than those of monoculture (27.97%, 29.61%) (*P*<0.05). Similarly, the protein content of the polyculture of *L. aequinoctialis+L. punctata*+*S. polyrhiza* at 20°C was 30.12%, significantly higher than those of monoculture (27.97%, 26.99%, 29.61%) (*P*<0.05). These findings indicated that the polyculture of duckweed species is favourable for protein accumulation at low temperature. Therefore, a mixed culture of duckweed is a feasible approach for protein production in the regions with lower temperature. At higher temperature (25 and 30°C), only the polyculture of *L. aequinoctialis* and *S. polyrhiza* at 30°C had a higher protein content than those of their monocultures, while the other polycultures showed median protein contents compared with their monocultures. This suggested that polyculture did not have a significant advantage over the monoculture in terms of protein content at higher temperature.

### Effect of light intensity on duckweed growth

Light intensity significantly affected the duckweed growth. In all cases of monoculture or polyculture, the highest relative growth rates and starch/protein content was achieved at the light intensity of 105 μmol·m^−2^·s^−1^, except for the relative growth rate of *S. polyrhiza* (Table 2).

The relative growth rate of *S. polyrhiza* increased as the light intensity increased from 30 to 75 μmol·m^−2^·s^−1^, but decreased at 105 μmol·m^−2^·s^−1^, suggesting that a lower irradiance is preferable for biomass accumulation of this duckweed isolate. As the light intensity increased from 30 to 105 μmol·m^−2^·s^−1^, the relative growth rate of *L. aequinoctialis* and *L. punctata* increased by almost 1.7-fold, from 0.11 to 0.19 day^−1^, and from 0.11 to 0.18 day^−1^, respectively. However, no significant increment was observed between the polycultures and their monocultures.

The duckweed accumulated more starch content as the light intensity increased (Table 2). Most of the polycultures showed median starch contents compared with their monocultures. But, under low light intensity (30 μmol·m^−2^·s^−1^), the polyculture of *L. aequinoctialis*, *L. punctata* and *S. polyrhiza* reached the highest starch content (7.71%), compared with those of monocultures (6.50%, 7.04%, 7.35%). In particular, the starch content of the polyculture of *L. aequinoctialis* and *L. punctata* was significantly higher than those of their monocultures under the light intensity of 105 μmol·m^−2^·s^−1^ (*P*<0.05). These results are in sharp contrast with the previous finding as described by Zhao et al. [18], where the polyculture (*L. minor* OT and *L. punctata* OT) tends to accumulate more starch at low irradiance. The isolates derived from their study were recovered from areas often covered by cloudy and rainy weather, whereas the isolates in the present study were obtained from areas with higher irradiance. Thus, the different performance of geographical isolates to irradiance might result from adaptation of duckweeds to local environment. In addition, these results indicated that polyculture of duckweed is a preferable method for starch production in areas with high irradiance.

The light intensity had an evident impact on protein content. The protein contents of duckweed in polycultures or monocultures were almost doubled as the light intensity increased from 30 to 105 μmol·m^−2^·s^−1^ (Table 2). Most of the protein content of the polycultures showed median protein contents compared with their monocultures. However, higher protein contents in polyculture than in monocultures were achieved by *L. aequinoctialis+L. punctata*+*S. polyrhiza* at 30 μmol·m^−2^·s^−1^ and by *L. punctata*+*S. polyrhiza* at 105 μmol·m^−2^·s^−1^. Particularly, a significant increase in protein content was observed between *L. aequinoctialis+S. polyrhiza* (33.00%) and their monocultures (29.53%, 32.60%) at 75 μmol·m^−2^·s^−1^ (*P*<0.05), suggesting that polyculture have a significant advantage over the monoculture in terms of protein content.

### Effect of N and P contents on duckweed growth

Nitrogen and phosphorus have been proved to be important factors for duckweed growth [26–28]. Nutrient conditions were separately tested on the isolates either in polyculture or in monocultures at different levels as described in the ‘Materials and methods’. In all cases of monoculture or polyculture, the relative growth rates and protein contents decreased as the concentrations of N and P deceased, while starch contents increased as the decrease in nutrients.

As shown in Table 3, the highest relative growth rates were achieved at the highest concentrations of N and P (35 mg·N·l^−1^ and 15 mg·P·l^−1^), suggesting that higher N and P concentrations were favourable for duckweed growth. However, at all nutrient concentrations, the polyculture did not show a significant advantage over monocultures in terms of relative growth rate.

**Table 3:** Relative growth rate, starch content and crude protein content of the duckweed in the mixture and monoculture in media with different concentration of N and P A=35 mg·N·l^−1^, 15 mg·P·l^−1^; B=3.5 mg·N·l^−1^, 1.5 mg·P·l^−1^; C=0 mg·N·l^−1^, 0 mg·P·l^−1^. The relative growth rate and starch/protein content were measured at 12 days after inoculation. Different lower-case letters in the same column denote significant differences according to *Duncan* test (*P*<0.05).

	Relative growth rate (day^−1^)			Starch content (% DW)			Crude protein content (% DW)		
Culture	A	B	C	A	B	C	A	B	C
*L. aequinoctialis*	0.19±0.0071a	0.10±0.0105a	0.08±0.0016a	12.49±0.2771a	21.70±0.1624a	28.83±0.1693a	25.82±0.2244a	15.98±0.0553a	11.99±0.0960a
*L. punctata*	0.19±0.0036a	0.09±0.0014a	0.07±0.0022a	13.40±0.2435^b^	22.63±0.0538^c^	32.82±0.0767^d^	31.91±0.3357^d^	18.93±0.0994^c^	14.43±0.1654^d^
*S. polyrhiza*	0.18±0.0109a	0.09±0.0064a	0.06±0.0028a	13.97±0.1347^d^	23.23±0.0854^d^	34.24±0.0521^f^	30.61±0.3159^c^	21.70±0.0904^d^	15.62±0.1050e
*L. aequinoctialis*+*L. punctata*	0.19±0.0069a	0.10±0.0046a	0.08±0.0029a	13.05±0.1311^b^	21.94±0.1834^b^	30.22±0.0306^b^	27.24±0.6978^b^	16.59±0.0968^b^	13.00±0.2010^b^
*L. aequinoctialis*+*S. polyrhiza*	0.19±0.0110a	0.09±0.0116a	0.08±0.0015a	13.65±0.1215^cd^	23.78±0.1950e	31.93±0.0846^c^	31.02±0.4856^b^	21.57±0.0384^d^	13.51±0.2233^c^
*L. punctata*+*S. polyrhiza*	0.18±0.0092a	0.09±0.0025a	0.06±0.0018a	13.58±0.0488^d^	22.81±0.0455^c^	33.72±0.0593e	32.41±0.1532^d^	21.66±0.0688^d^	15.82±0.1652e
*L. aequinoctialis*+*L. punctata*+*S. polyrhiza*	0.19±0.0142a	0.10±0.0018a	0.07±0.0021a	13.21±0.1876^bc^	23.15±0.0897^d^	34.14±0.1550^f^	31.12±0.2671^c^	19.16±0.1971^c^	14.75±0.0482^d^

It is well-known that nutrient starvation can induce starch accumulation in duckweed [29,30]. Our results are consistent with this finding. The starch contents of every combination were doubled by more than 2-folds as the N and P concentrations decreased from 35 mg·N·l^−1^ and 15 mg·P·l^−1^ to 0 mg·N·l^−1^ and 0 mg·P·l^−1^ (Table 3). The majority of the starch contents of the polycultures showed median starch contents compared with their monocultures. But a significant increase in starch content was observed between *L. aequinoctialis+S. polyrhiza* (23.78%) and their monocultures (21.70%, 23.23%) at a concentration of 3.5 mg·N·l^−1^ and 1.5 mg·P·l^−1^ (*P*<0.05). Furthermore, the starch content of the polyculture of three species in B (low nutrient concentration) or C medium (nutrient starved) were almost equal to the highest one of monocultures (Table 3). All these results suggested that polyculture could promote the population to accumulate starch.

Unlike the starch content, the protein content decreased as N and P concentrations decreased (Table 3). *S. polyrhiza* showed the highest protein content among the three species. Although the majority of the protein content of the polycultures showed median protein contents compared with their monocultures, a significant increase in protein content was observed between *L. aequinoctialis+S. polyrhiza* (32.68%) and their monocultures (26.60%, 31.29%) at high concentration of N and P (*P*<0.05). Interestingly, the protein content of the polyculture of *L. punctata* and *S. polyrhiza* (21.66%) at low nutrient concentration (B medium) were almost equal to the highest one of monocultures (*S. polyrhiza*, 21.70%), but the polyculture at a lower concentration (C medium) achieved the highest protein content (15.82%), as compared with monocultures (14.43%, 15.62%). These findings indicated that the polyculture can promote population to accumulate protein at low nutrient concentration and proper combination of duckweed species can achieve more protein at high nutrient concentration.

### Starch/protein production

The most important applications of duckweed biomass are the high starch yield as feedstock for biofuels and the high protein yield for animal feed. The starch/protein productivity of duckweed depends on its content and biomass production. In the present study, the starch/protein productivity was calculated by its content and biomass production. The results showed that the highest starch productivity under different culture conditions was all achieved by polyculture (Table 4), namely *L. punctata+S. polyrhiza* (16.66 g·m^−2^, 25°C), *L. aequinoctialis+L. punctata* (19.01 g·m^−2^, 105 μmol·m^−2^·s^−1^) and *L. aequinoctialis+L. punctata+S. polyrhiza* (28.78 g·m^−2^, medium C). And the starch productivity of these polycultures was significantly higher than those of their monocultures, suggesting that proper polyculture of duckweed species could enhance starch production. The polyculture of *L. punctata* and *S. polyrhiza* achieved the highest protein productivity at 25°C (37.55 g·m^−2^) and at light intensity of 105 μmol·m^−2^ s^−1^ (49.86 g·m^−2^), respectively (Table 5). Under different concentration of N and P, the monoculture of *L. punctata* showed the highest protein productivity (36.14 g·m^−2^) in medium A, indicating that polyculture did not show advantage over monoculture in terms of protein production at high concentration of N and P.

**Table 4:** Starch production (g·m^−2^) of the duckweed in the mixture and monoculture under different culture conditions A=35 mg·N·l^−1^, 15 mg·P·l^−1^; B=3.5 mg·N·l^−1^, 1.5 mg·P·l^−1^; C=0 mg·N·l^−1^, 0 mg·P·l^−1^. Different lower-case letters in the same column denote significant differences according to *Duncan* test (*P*<0.05).

	Temperature (°C)			Light intensity (μmol·m^−2^·s^−1^)			Concentration of N and P		
Culture	20	25	30	30	75	105	A	B	C
*L. aequinoctialis*	14.22±0.2976^ab^	15.11±0.1840a	11.09±0.1154^bc^	3.02±0.1298^b^	13.09±0.4811^abc^	16.27±0.3514^b^	15.02±0.2801^b^	23.41±0.3187d	25.66±0.2622^c^
*L. punctata*	12.16±0.3860a	15.03±0.4631a	10.17±0.4210^ab^	3.00±0.0963^b^	13.68±0.5538^bc^	17.78±0.1976^cd^	14.87±0.3730^b^	21.87±0.2739c	26.00±0.5951^c^
*S. polyrhiza*	13.18±0.1735a	14.76±0.2781a	9.67±0.3330a	2.62±0.1466a	12.60±0.2779a	11.92±0.2898a	13.99±0.1534a	20.37±0.1779a	20.85±0.4570a
*L. aequinoctialis*+*L. punctata*	15.46±0.3861^c^	14.81±0.3949a	10.67±0.1382^abc^	3.08±0.1114^b^	14.70±0.1764^d^	19.01±0.4131e	14.98±0.2236^b^	22.80±0.3434d	28.58±0.7542^d^
*L. aequinoctialis*+*S. polyrhiza*	14.73±0.5791^bc^	14.97±0.1393a	12.80±0.4170^d^	3.03±0.2436^b^	12.92±0.7243^ab^	18.32±0.1435^de^	15.07±0.1005^b^	22.37±0.2396d	28.33±0.8915^d^
*L. punctata*+*S. polyrhiza*	13.80±0.3876^ab^	16.66±0.2589^b^	10.66±0.5342^abc^	3.29±0.2511^b^	13.73±0.3557^bc^	17.09±0.2057^bc^	15.26±0.2714^b^	21.27±0.1986b	22.35±0.6746^b^
*L. aequinoctialis*+*L. punctata*+*S. polyrhiza*	13.96±0.2604^ab^	15.57±0.4432a	11.97±0.3210^cd^	3.32±0.1202^b^	13.85±0.4386^c^	16.67±0.5601^bc^	15.13±0.3193^b^	25.50±0.6365e	28.78±0.1241^d^

**Table 5:** Crude protein production (g·m^−2^) of the duckweed in the mixture and monoculture under different culture conditions A=35 mg·N·l^−1^, 15 mg·P·l^−1^; B=3.5 mg·N·l^−1^, 1.5 mg·P·l^−1^; C=0 mg·N·l^−1^, 0 mg·P·l^−1^. Different lower-case letters in the same column denote significant differences according to *Duncan* test (*P*<0.05).

	Temperature (°C)			Light intensity (μmol·m^−2^·s^−1^)			Concentration of N and P		
Culture	20	25	30	30	75	105	A	B	C
*L. aequinoctialis*	28.91±0.5437^d^	39.49±0.6432^c^	25.69±0.7280^b^	7.50±0.0672^c^	33.34±0.4140^c^	41.74±0.1344^d^	32.31±0.1951^ab^	17.23±0.1018a	10.67±0.0866^b^
*L. punctata*	23.16±0.6364^ab^	35.75±0.9701a	24.14±0.7709^ab^	6.72±0.0946^b^	12.94±0.1251^b^	37.06±0.1418^b^	35.84±0.1582^c^	18.30±0.0297^b^	11.44±0.0346^c^
*S. polyrhiza*	22.73±0.5918a	38.26±0.8031^bc^	23.08±0.5067a	6.12±0.0501a	29.67±0.1248a	26.96±0.1389a	31.60±0.0787a	19.03±0.0426^c^	9.51±0.0179a
*L. aequinoctialis*+*L. punctata*	28.20±0.5395^cd^	36.38±0.2534a	24.73±0.7218^b^	7.32±0.0744^c^	33.86±0.14671^c^	39.86±0.3874^c^	32.91±0.0856^b^	17.24±0.1097a	12.29±0.0168^d^
*L. aequinoctialis*+*S. polyrhiza*	29.15±0.6453^d^	37.82±0.5435^b^	30.45±0.4748^d^	7.33±0.0897^c^	36.10±0.2171^d^	42.69±0.3594^d^	35.88±0.0766^c^	21.20±0.1112e	11.98±0.0419^cd^
*L. punctata*+*S. polyrhiza*	24.34±0.7955^b^	38.71±0.0438^bc^	24.96±0.1343^b^	7.40±0.0767^c^	31.89±0.1959^b^	49.86±0.2406e	36.76±0.0304^c^	20.19±0.0246^d^	10.49±0.0232^b^
*L. aequinoctialis*+*L. punctata*+*S. polyrhiza*	27.26±0.5594^c^	37.76±0.5171^b^	28.78±0.6347^c^	7.59±0.0963^c^	33.26±0.2506^c^	38.47±0.2687^bc^	36.01±0.1208^c^	21.10±0.0575e	12.44±0.0462^d^

Overall, to mix duckweed species in culture is a useful approach to increase the production of starch and crude protein. The enhanced biomass productivity in polyculture could be shaped by several possible processes. First, polycultures may promote tolerance to or resilience from environmental disturbance and enhance resistance to disease and pest damage [31]. Second, improved plant performance by genetic diversity may occur via selection effects, whereby diverse populations have a higher probability of containing high performance genotype, or complementarity effect, whereby niche differentiation, facilitation or counteraction among genotypes results in increased polyculture performance [32]. Third, different genotypes may differ in their resource use (e.g. uptake of N and P) or facilitate each other and thus the better performance in patches with high genotypic diversity can be due to higher resource uptake or facilitation [33]. However, the exact nature of the positive relationship in polyculture is still unclear. Such information is important and can provide useful insights into conservation and restoration strategies of duckweed at the population level. This mechanism will need to be investigated carefully in future work.

## CONCLUSIONS

Temperature, light intensity and concentration of N and P significantly affect duckweed biomass accumulation. Different polycultures varied in biomass production. As compared with monoculture, all the polycultures showed a median relative growth rate, while the majority of the polycultures showed a median starch content or protein content. But, proper polyculture of duckweed species can significantly enhance the starch/protein content, and finally generate higher starch or protein production. The present study provides useful references for future large-scale duckweed cultivation.

## Abbreviations

DW: dry weight
